# Association of iron supplementation and dietary diversity with nutritional status and learning outcomes among adolescents: Results from a longitudinal study in Uttar Pradesh and Bihar, India

**DOI:** 10.7189/jogh.11.04037

**Published:** 2021-07-24

**Authors:** Sachin Shinde, Isabel Madzorera, Wafaie W Fawzi

**Affiliations:** 1 Department of Global Health and Population, Harvard T.H. Chan School of Public Health, Boston, Massachusetts, USA; 2 Center for Inquiry into Mental Health, Pune, India; 3 Department of Epidemiology, Harvard T.H. Chan School of Public Health, Boston, Massachusetts, USA; 4 Department of Nutrition, Harvard T.H. Chan School of Public Health, Boston, Massachusetts, USA

## Abstract

**Background:**

Poor nutritional status and educational outcomes are associated with adverse health consequences throughout the life course.

**Objectives:**

We examined the associations of sex, iron and folic acid (IFA) supplementation, deworming, and dietary diversity with nutritional status and learning outcomes among Indian adolescents.

**Methods:**

Data of 12 035 adolescents from two rounds (2015-16 and 2018-19) of the Understanding the Lives of Adolescents and Young Adults surveys in Uttar Pradesh and Bihar, India were used. Multivariate linear and logistic regression models were used to estimate the prospective relationships of explanatory variables in the first round of the survey with nutritional status (ie, hemoglobin, BMI- and height-for-age *z*-scores, and incidence of anemia, stunting, and underweight) and learning outcomes (ie, reading proficiency, math proficiency, and school dropout) in the second round of the survey. The multivariable models were adjusted for a range of confounders, clustering by the population sampling unit and sampling weights.

**Results:**

Girls had a lower hemoglobin (adjusted mean difference (aMD) = -2.07; 95% confidence interval (CI) = -2.20, -1.94; *P* < 0.001) and height-for-age *z*-score (aMD = -0.45; 95% CI = -0.53, -0.38; *P* < 0.001) and higher BMI-for-age *z*-score (aMD = 0.36; 95% CI = 0.27, 0.44; *P* < 0.001) compared to boys. Girls had poorer reading (adjusted odds ratio (aOR) = 0.81; 95% CI = 0.70, 0.95; *P* = 0.01) and math proficiency (adjusted odds ratio aOR = 0.36; 95% CI = 0.31, 0.42; *P* < 0.001), and increased risk of school dropout (aOR = 1.65; 95% CI = 1.40, 1.95; *P* < 0.001) than boys. Receiving IFA tablets was not significantly associated with hemoglobin levels of adolescents overall, however we noted a significant beneficial relationship among girls (aMD = 0.41; 95% CI = 0.00, 0.82) but not among boys (aMD = 0.02; 95% CI = -0.38, 0.43) (*P* for interaction = 0.09). Receiving IFA tablets was positively associated with reading proficiency (aOR = 1.47; 95% CI = 1.07, 2.01; *P* = 0.02), math proficiency (aOR = 1.51; 95% CI = 1.16, 1.98; *P* < 0.001) and a reduced risk of school dropout (aOR = 0.72; 95% CI = 0.54, 0.96; *P* = 0.03). Deworming was not associated with nutritional status and learning outcomes. Diverse dietary intake was positively associated with hemoglobin, height-for-age *z*-score and math proficiency, and a reduced risk of school dropout in adolescents.

**Conclusion:**

Integrated nutritional and health interventions and strengthening the existing nutrition supplementation program are critical to address adolescents’ nutritional needs and improve educational outcomes.

Adolescence is the second-fastest growth stage in life after infancy. It is accompanied by a growth spurt, neurocognitive maturity, and social role transitions, with important consequences for nutritional needs. However, adolescents globally suffer from a high prevalence of macro-and micro-nutrient deficiencies and their consequences [1], which often manifest as nutritional anemia, underweight, stunting, or overweight/obesity; and, lead to many chronic diseases [2]. Malnutrition among adolescents also leads to impaired motor and cognitive development, low intellectual quotient, and poor academic performance [1]. A bi-directional relationship exists between nutrition and health outcomes on one hand and educational outcomes on the other [3]. Healthy young people learn better and have improved life chances, and more-educated young people have better nutrition and health outcomes.

India is home to 253 million adolescents, accounting for 21% of its population [4]. The National Family Health Survey (NFHS-4) in India reported a prevalence of anemia among girls aged 15-19 years of 54.0% compared to a prevalence of 29.0% among boys of the same age group [5]. A pooled analysis of 2146 population-based studies of 128.9 million children, adolescents, and adults worldwide noted the highest prevalence of moderate to severe underweight in children and adolescents in South Asia; with one in five girls aged 5-19 years and nearly one-third of their male peers being underweight. The lowest BMIs for both male and female adolescents were noted in South Asia, including India [6].

The etiology of anemia is multi-faceted and includes micronutrient deficiencies, chronic infections, and inherited hemoglobin disorders; however, approximately 50% of the anemia burden is estimated to be related to iron deficiency [7]. Iron and folate deficiencies are among the top risk factors of disability-adjusted years worldwide [1,8]. The rapid growth that occurs during adolescence, the onset of menstruation among adolescent girls, the consumption of predominantly plant-based diets with high phytate levels and low bioavailable iron, and limited intake of animal source foods (sources of bioavailable heme iron), all contribute to low iron stores and increase the susceptibility of adolescents to anemia in developing countries [9].

In response to high levels of iron deficiency anemia, the government of India launched a nationwide weekly iron and folic acid (IFA) supplementation and bi-annual deworming program for all age groups in January 2011 [10]. Adolescents aged 10-19 years are provided with a weekly oral dose of 100 mg elemental iron and 500 μg folic acid throughout the year. A limited number of Indian studies of IFA supplementation suggest a significant reduction in anemia and improvement in cognitive ability and intelligence quotient, and no effects on academic achievement, memory, and motor development [11-14]. However, these studies were mostly carried out in school-going girls and the impact of IFA supplementation for adolescent boys is not clear. Additionally, data on the coverage and effects of universal IFA supplementation in adolescent boys and girls are limited in India [15].

Over one-fifth of the Indian population suffers from chronic hunger due to various factors, including poverty, food shortages and waste, limited nutritional quality, and unstable markets [16]. Limited dietary diversity can contribute to inadequate micronutrient intakes, which are vital for the growth and development of adolescents. Multiple studies have shown that higher dietary diversity is positively associated with better nutritional status and cognitive abilities of children [17-20]; however, this association is rarely studied in adolescents. Furthermore, studies conducted in Indian adolescents rarely collect longitudinal information on dietary diversity, overall diet quality, and micronutrient intake, and often suffer from a small sample size, narrow age inclusion, and omission of factors that may contribute to nutritional status and learning outcomes [21].

The “Understanding the Lives of Adolescents and Young Adults” (UDAYA) project in Uttar Pradesh and Bihar, India [22] provided a unique opportunity to fill the evidence gap on the relationships between adolescent nutritional supplementation, deworming, and dietary diversity with nutritional status and learning outcomes through a longitudinal study. The objectives of this paper were to 1) describe adolescent nutrition in terms of hemoglobin level, BMI- and height-for-age *z*-scores, the incidences of anemia, underweight, and stunting, and academic outcomes, including reading and math proficiency, and school dropout in northern India in the second round of UDAYA survey; and 2) examine the prospective relationships between receiving IFA tablets, deworming, and dietary intake in the first round of UDAYA survey with these outcomes in the second round of the survey.

## MATERIALS AND METHODS

### Data source and study population

Data were obtained from adolescents who participated in the two rounds of surveys of the Population Council’s UDAYA project in Uttar Pradesh and Bihar states of India [22]. Field workers collected data, using the computer-assisted personal interviewing technique, from 20 589 unmarried male and female adolescents aged 10-19 years and married female adolescents aged 15-19 years in the first round of the survey in 2015-16 and re-interviewed 16 292 adolescents in the second round of the survey in 2018-19.

The study sample for nutrition outcomes included 3375 unmarried male and female adolescents aged 13-19 years in 2018-19 with an assessment of hemoglobin and anthropometric measures (ie, height and weight) in both rounds of the surveys. We excluded those participants who were aged 20-22 years in 2018-19 from anthropometric outcome analyses due to the unavailability of standardized, age-specific cut-offs for this group. Trained field investigators measured hemoglobin in grams per deciliter (g/dL) of whole blood using a finger-prick blood sample and portable Hemocue Hb 201+ analyzer instrument. Height was measured to the nearest 0.1 cm using a SECA-213 stadiometer and weight was measured to the nearest 100 g using a SECA-874 electronic scale.

The sample for learning outcomes (ie, reading proficiency, math proficiency, and school dropout) included 12 035 unmarried adolescents aged 13-22 years in 2018-19. Reading and math proficiencies were assessed by the Annual Status of Education Report tools, used to estimate basic learning levels of Indian school-going children since 2005 [23]. Table S1 in the **Online Supplementary Document** provides further details on the interviews conducted and anthropometric assessments completed with adolescents in both rounds of surveys and samples in the analyses.

### Study variables

#### Exposure variables

Information on socioeconomic variables, including sex, was obtained through a structured, standardized questionnaire. Exposure to the IFA supplementation and deworming program was assessed through closed-ended questions in the first round of the survey in 2015-16. Adolescents were asked whether they received IFA and/or de-worming tablets in the last year from school or frontline health workers in the community. In the dietary portion of the questionnaire, adolescents provided frequency of consumption of foods from the following food groups in the first round of the survey: (1) pulses/beans, (2) dark green vegetables, (3) other vegetables, (4) fruits, (5) eggs, (6) meat/poultry, (7) fish and seafood, and (8) milk and milk products. Several examples of food items were provided during the interview to describe each food group. Responses were coded into four categories of frequency: daily, weekly, occasionally (less than once a week), and never. Dietary scores were calculated as the number of food groups adolescents reported consuming daily (score range from 1 to 8) using the method described by the Food and Agriculture Organization (FAO) of the United Nations for Household and Individual Dietary Diversity [24]. The dietary scores were divided into quartiles for analysis.

#### Nutrition outcomes

We examined the following nutrition outcomes in the second round of the survey: hemoglobin, BMI- and height-for-age *z*-scores, and incidence of anemia, underweight, and stunting. Standard age- and sex-specific World Health Organization (WHO) cut-offs were applied to define anemia (ie, 10 or less g/dL for 13 to 14 year-old boys and 13 to 19 year-old girls and 12 or less g/dL for boys in ages1519) [25]. BMI was calculated as weight in kilograms divided by height in meters squared and rounded to one decimal place. BMI- and height-for-age *z*-scores were calculated using the WHO standard for children aged 5-19 years [26,27]. Following the WHO classifications for adolescents, underweight was defined as a BMI *z*-score for age and sex<-2 SD [26,27]. Stunting was defined as a height *z*-score for age and sex<-2 SD [26,27].

#### Academic outcomes

The reading component assessed four levels of proficiency in the Hindi language: the ability to recognize letters, read words, read a short paragraph (grade 1 level text; ages 6-7 years), and read a ‘story’ (grade 2 level text; ages 7-8 years). The math component assessed four levels of proficiency: the ability to recognize single-digit numbers, double-digit numbers, solve two-digit subtraction problems, and three-digit division problems. School dropout status in 2018-19 was determined through a response to a closed-ended question on school enrolment status.

#### Confounding variables

We selected confounders for the regression analysis based on the literature on risk factors for adolescent nutrition [3] and the availability of data in the first round of the UDAYA survey. Broadly, we included variables that covered demographic, parental, and social factors and depressive symptoms. Demographic factors included respondent’s age, religion, caste, household wealth, and mother’s education. A household wealth index was constructed using household asset data on ownership of selected durable goods, means of transportation, as well as data on access to several amenities (ie, type of house, agricultural land owned, irrigated land owned, access to toilet facility, cooking fuel used, access to drinking water facility, access to electricity, and ownership of household assets). Based on the ownership of assets, an index score was constructed. Households were then ranked according to the index score and divided into quintiles, with the first quintile representing households with the poorest wealth status and the fifth quintile representing households with the wealthiest status.

Parental factors included exposure to parental violence, the experience of gender discrimination within the family, and discussion with parents on physical change and school performance. Participants responded to a closed-ended question whether they had ever witnessed incidents where their father had beaten their mother. Adolescents responded to three questions with closed-ended responses on whether their parents ever treated opposite-sex siblings differentially when providing food (amount or quality of), education (duration and quality of), and pocket money, and the questions were combined to assess the experience of gender discrimination within family. Participants also responded to two closed-ended questions on whether they ever discussed school performance and discussed physical changes in the body (eg, voice change, facial hair growth, etc.) with their mother and/or father in the past year at the first survey.

Psychosocial factors considered included adolescents’ experience of sexual abuse, their total number of friends, their use of substances (including tobacco, alcohol, and other drugs), and whether they received a ration or food from the Anganwadi center. Psychosocial factors including exposure to violence and abuse are associated with anemia and underweight among adolescents [28,29]. We included these covariates primarily because psychological stress causes higher levels of oxidative stress and metabolic stress, both of which can contribute to anemia and malnutrition [28]. The experience of sexual abuse was assessed by whether adolescents were ever deliberately touched on their private parts when they did not want to be touched. Adolescents responded to three questions with closed-ended responses on whether they have ever used tobacco or tobacco products, alcohol, and other substances to assess the use of substances in the first survey round. We also assessed whether the adolescents were engaged in paid work outside of school in the past year. Experiencing mental health problems such as depression and anxiety can also be a risk factor for involuntary weight loss or gain in young people and adults and hence included as a covariate [30]. Depressive symptoms were measured with the Patient Health Questionnaire (9-item version), on which the total score ranges from 0 to 27 with a higher score indicating more severe symptoms [31,32].

### Statistical analysis

Statistical analyses were performed using Stata 14. To examine and summarize the data, sample characteristics were described using frequencies for categorical variables, and means, and standard deviations for continuous variables. To estimate the associations between the exposure variables and continuous outcomes, we used multivariate linear regression and reported adjusted mean difference (aMD) with 95% confidence intervals (CIs). For binary outcomes, we estimated the adjusted odds ratio (aOR) with 95% CIs, using logistic regression. All crude and multivariable models were adjusted for clustering by the population sampling unit and sampling weights. The multivariable models for nutrition outcomes including hemoglobin, BMI- and height-for-age z-scores, and anemia accounted for respondent’s sex, age, state, residence, caste, household wealth index quintile, dietary score quartile, received IFA tablets, deworming control, engagement in paid work, and whether adolescent received ration or cooked meal from the Anganwadi center. The multivariable model for stunting controlled for respondents’ sex, age, caste, state, residence, household wealth index quintile, mother’s education, dietary score quartile, received IFA tablets, deworming control, engagement in paid work, the experience of gender discrimination within family, and discussion on physical change with parents. The multivariable model for underweight adjusted for respondents’ sex, age, religion, caste, state, household wealth index quintile, mother’s education, dietary score quartile, received IFA tablets, deworming control, engagement in paid work, experience of sexual abuse, and discussion on physical change with parents. All multivariable models for learning outcomes accounted for respondent’s sex, age, religion, caste, state, residence, household wealth index quintile, mother’s education, dietary score quartile, received IFA tablets, deworming control, engagement in paid work, experience of sexual abuse, number of friends, ever used substances, depressive symptoms, exposure to parental violence, discussion on school performance with parents and experience of gender discrimination within the family. We also examined the interactions of sex with receiving IFA supplementation, deworming, or dietary score quartile in their associations with continuous outcomes ie, hemoglobin, BMI- and height-for-age z-scores. We accounted for missing covariate data with the missing indicator method [33]. Covariates with missing values were replaced with one value (ie, ‘9’) and included in the crude and multivariate models as one of the categories of the variable.

### Ethical considerations

During each round of the survey, all participating adolescents were provided with detailed information about the study before inviting them to participate. For all minor participants (less than 18 years old), parental written consent and participant assent were sought. For all participants aged 18 years and above, written consent was obtained. The Institutional Review Board of the Population Council approved both the rounds of surveys.

## RESULTS

Data from 12 035 adolescents who participated in the two surveys were analyzed. The study sample consisted of 63.2% girls and 36.8% boys (**Table 1**). On average, boys were slightly younger (14.9 ± 2.7 years) than girls (15.8 ± 2.3 years). Adolescent boys and girls in the study were mostly Hindu and from backward castes. A small percentage of adolescents received IFA supplementation and deworming tablets. More girls than boys received IFA supplementation (4.6% vs 2.8%) and deworming tablets (17.3% vs 12.8%). The mean (±SD) dietary score was slightly higher among boys (2.1 ± 1.1) than girls (2.0 ± 1.1). More boys than girls were engaged in paid work in the last 12 months, whereas more girls than boys experienced gender discrimination within the family. Table S2 in the **Online Supplementary Document** shows the percentages of adolescent boys and girls who consumed food items from the pulses and beans, dairy products, dark green vegetables, other vegetables, fruits, eggs, poultry items, fish, and milk and milk products groups. The three food groups most consumed by adolescent boys and girls in the study were other vegetables, pulses and beans, and milk and milk products.

**Table 1:** Background characteristics of 12 035 adolescent girls and boys who participated in the two rounds of UDAYA surveys in Uttar Pradesh and Bihar, India

	Girls (N = 7607)	Boys (N = 4428)	Total (N = 12035)
Mean age (in years; SD)	15.77 (±2.23)	14.85 (±2.68)	15.43 (±2.45)
State:			
Bihar	45.64%	48.06%	46.53%
Uttar Pradesh	54.36%	51.94%	53.47%
Residence:			
Rural	54.99%	55.08%	55.02%
Urban	45.01%	44.92%	44.98%
Religion:			
Hindu	74.59%	84.24%	78.14%
Others*	25.41%	15.76%	21.86%
Caste†			
Scheduled castes	20.80%	23.30%	21.72%
Scheduled tribes	0.54%	1.21%	0.79%
Other backward castes	56.68%	56.52%	56.62%
General castes	21.84%	18.90%	20.76%
Missing	0.14%	0.04%	0.11%
Mother’s education:			
No schooling	66.04%	64.93%	65.63%
1-7 years of education	10.94%	11.36%	11.09%
8-9 years of education	8.73%	9.49%	9.01%
10 or more years of education	14.29%	14.23%	14.27%
Received IFA supplementation in last 12 months	4.68%	2.89%	4.02%
Received deworming control tablets in last 12 monts	17.32%	12.78%	14.45%
Mean Dietary score (SD; score range: 0-8)‡	1.96 (±1.06)	2.05 (±1.09)	1.99 (±1.07)
Received ration or meal from Anganwadi Centre	2.41%	2.08%	2.29%
Engaged in paid work in the last 12 months	16.05%	21.48%	18.05%
Number of friends			
No friends	4.15%	2.30%	3.47%
1-2	36.41%	30.78%	34.34%
3 or more	59.43%	66.92%	62.19%
Substance use	1.45%	13.98%	6.06%
Experience of sexual abuse	7.99%	1.49%	5.60%
Mean depressive symptoms (SD; score range: 0-27)§	2.16 (±3.95)	1.28 (±2.84)	1.83 (±3.51)
Discussed school performance with parents	59.70%	66.62%	62.24%
Discussed physical change with parents	62.01%	5.71%	41.30%
Exposure to parental violence	24.24%	17.91%	21.91%
Experience of gender discrimination within the family	12.30%	7.07%	10.38%

IFA – Iron and Folic Acid, SD – standard deviation

*Others include Muslim, Christian, Buddhist/NeoBuddhist, Sikh, Jain, and no specified religion.

†In the caste categories, backward or other caste is a collective term used by the Government of India to classify socially disadvantaged castes, while the scheduled castes are officially designated groups of historically disadvantaged people in India. Scheduled tribes are officially designated tribes, tribal communities, parts of, or groups within such tribes. General caste is a term used in India to denote groups of people who do not qualify for any of the affirmative action schemes by the Government of India.

‡Food groups include pulses, leafy vegetables, other vegetables, fruits, milk and milk products, eggs, fish, and meat. A higher score indicates better dietary diversity.

§A higher score indicates more severe depressive symptoms.

The absolute burden of nutritional and learning outcomes was high among the study participants. The mean (±SD) hemoglobin was 12.3 g/dL (±1.9), and relatively higher among boys (13.3 g/dL ±1.7) compared to girls (11.2 g/dL ±1.6). The incidence of anemia was 12.7% and the incidence of stunting was 10.2%, and both were higher in girls than boys (17.8% vs 8.3% and 12.3% vs 5.6%; respectively). The incidence of underweight was 5.8% and slightly higher among boys compared to girls (7.0% vs 4.6%). The reading and math proficiency was poor (72.8% and 46.4% respectively) and school dropout rates were high (27.9%) among the study participants. Reading proficiency skills of girls and boys were similar (72.2% vs 73.7%; respectively) however, girls had poorer math proficiency skills (39.9% vs 56.9%) and a higher rate of school dropout (31.2% vs 22.9%) than boys.

**Table 2** shows the unadjusted and adjusted associations between sex and nutrition status and learning outcomes. Girls, compared to boys, were at an increased risk of anemia (aOR = 2.58; 95% CI = 1.87, 3.55; *P* < 0.001). Being a female adolescent was associated with lower hemoglobin and height-for-age *z*-score, and higher BMI-for-age *z*-score. There was no statistically significant association between sex and stunting or underweight in adolescents. Girls were likely to have lower reading (aOR = 0.81; 95% CI = 0.70, 0.95; *P* = 0.01) and math (aOR = 0.36; 95% CI = 0.31, 0.42; *P* < 0.001) proficiency and a higher risk for school dropout (aOR = 1.65; 95% CI = 1.40, 1.95; *P* < 0.001) compared to boys.

**Table 2:** Associations between sex and nutrition and learning outcomes in adolescent boys and girls in Uttar Pradesh and Bihar, India (2015-16 to 2018-19)

Continuous outcomes	Girls (N = 1609), mean (SD)	Boys* (N = 1766), mean (SD)	Unadjusted β† (95% CI)	Adjusted β† (95% CI)
Hemoglobin (g/dl)‡	11.23 (1.59)	13.32 (1.69)	-2.09 (-2.21, -1.96)‡‡	-2.07 (-2.20, -1.94)‡‡
BMI-for-age *z*-score‡	-0.74 (1.00)	-1.14 (1.16)	0.39 (0.31, 0.47)‡‡	0.36 (0.27, 0.44)‡‡
Height-for-age *z*-score‡	-1.63 (0.92)	-1.12 (1.02)	-0.51 (-0.58, -0.44)‡‡	-0.45 (-0.53, -0.38)‡‡
**Binary outcomes**	**(n/N)**§	**(n/N)**§	**Unadjusted odds ratio† (95% CI)**	**Adjusted odds ratio† (95% CI)**
Anemia‡	252/1414	137/1657	2.63 (1.94, 3.58)‡‡	2.58 (1.87, 3.55)‡‡
Stunting¶	149/1207	133/1553	1.15 (0.84, 1.58)	1.07 (0.73, 1.56)
Underweight**	67/1444	100/1422	0.74 (0.47, 1.15)	0.89 (0.52, 1.51)
Reading proficiency††	5163/7147	3211/4354	0.93 (0.82, 1.07)	0.81 (0.70, 0.95)§§
Math proficiency††	2853/7147	2481/4354	0.44 (0.39, 0.49)‡‡	0.36 (0.31, 0.42)‡‡
School dropout††	1751/5618	852/3726	1.65 (1.44, 1.88)‡‡	1.65 (1.40, 1.95) ‡‡

SD – standard deviation, CI – confidence interval

*Reference category for all models.

†All unadjusted and adjusted models control for sampling unit–level clustering and sampling weights.

‡Adjusted for age, caste, state, residence, household wealth index quintile, mother’s education, dietary score, receiving IFA

§Number of adolescent boys and girls (n) in the sample (N).

supplementation, deworming control, engagement in paid work, and received ration or cooked meal from Anganwadi center.

¶Adjusted for age, caste, state, residence, household wealth index quintile, mother’s education, dietary score, receiving IFA supplementation, deworming control, engagement in paid work, experience of gender discrimination within family, and discussion on physical change with parents.

*Adjusted for age, religion, caste, state, household wealth index quintile, mother’s education, dietary score, receiving IFA supplementation, deworming control, engagement in paid work, experience of sexual abuse, and discussion on physical change with parents.

††Adjusted for age, religion, caste, state, residence, household wealth index quintile, mother’s education, dietary score, receiving IFA supplementation, deworming control, engagement in paid work, experience of sexual abuse, number of friends, ever used substances, depressive symptoms, exposure to parental violence, discussion on school performance with parents and experience of gender discrimination within the family.

‡‡*P* < 0.001.

§§*P* < 0.05.

We found no significant association between receiving IFA supplementation and adolescent BMI- and height-for-age *z*-scores, anemia, stunting, and underweight (**Table 3**). There was an association between receiving IFA supplementation and hemoglobin however, this was not statistically significant (aMD = 0.21; 95% CI = -0.07, 0.50; *P* = 0.14). Receiving IFA supplementation was positively associated with hemoglobin among girls but not boys (p for interaction = 0.09) (Table S1 in the **Online Supplementary Document**). Girls who received IFA supplementation were likely to have higher hemoglobin than those girls who did not receive it (aMD = 0.41; 95% CI = 0.00, 0.82) whereas there was no statistically significant difference between the mean hemoglobin of boys who received IFA supplementation and boys who did not receive it (aMD = 0.02; 95% CI = -0.38, 0.43). We also noted an apparent interaction between receiving IFA supplementation and adolescents’ height-for-age *z*-score (p for interaction = 0.06). Boys who received IFA supplementation were likely to have higher height-for-age *z*-score than boys who did not receive it (aMD = 0.26; 95% CI = 0.01, 0.51) while there was no statistically significant difference between the height-for-age *z*-score of girls who received IFA supplementation and those who did not receive it (aMD = -0.11; 95% CI = -0.29, 0.07). There was no association between the interaction of receiving IFA supplementation and sex with height-for-age *z*-score (*P* for interaction = 0.88) (Table S3 in the **Online Supplementary Document**).

**Table 3:** Associations between receiving IFA supplementation and nutrition and learning outcomes in adolescent boys and girls in Uttar Pradesh and Bihar, India (2015-16 to 2018-19)

Continuous outcomes	Received IFA supplementation,00 mean (SD), N = 143	No IFA supplementation*, mean (SD), N = 3232	Unadjusted β†, (95% CI)	Adjusted β†, (95% CI)
Hemoglobin (g/dL)‡	12.30 (1.83)	12.32 (1.95)	-0.04 (-0.35, 0.26)	0.21 (-0.07, 0.50)
BMI-for-age z-score‡	-1.03 (1.06)	-0.95 (1.11)	-0.07 (-0.26, 0.11)	-0.08 (-0.27, 0.10)
Height-for-age z-score‡	-1.35 (1.01)	-1.36 (1.01)	0.01 (-0.15, 0.18)	0.06 (-0.08, 0.21)
**Binary outcomes**	**(n/N)**§	**(n/N)**§	**Unadjusted odds ratio**† **(95% CI)**	**Adjusted odds ratio†** **(95% CI)**
Anemia‡	18/132	371/2939	1.11 (0.58, 2.15)	0.99 (0.49, 1.97)
Stunting¶	9/110	273/2650	2.33 (0.88, 6.15)	0.45 (0.17, 1.19)
Underweight**	7/122	160/2744	1.11 (0.51, 2.39)	1.19 (0.50, 2.85)
Reading proficiency††	371/479	8003/11 022	1.74 (1.25, 2.42)‡‡	1.47 (1.07, 2.01)§§
Math proficiency††	242/479	5092/11 022	1.45 (1.08, 1.94)§§	1.51 (1.16, 1.98)§§
School dropout††	120/439	2483/8905	0.78 (0.60, 1.02)‡‡	0.72 (0.54, 0.96)§§

IFA – iron and folic acid, SD – standard deviation, CI – confidence interval

*Reference category for all models.

†All unadjusted and adjusted models control for sampling unit–level clustering and sampling weights.

‡Adjusted for age, sex, caste, state, residence, household wealth index quintile, mother’s education, dietary score, deworming control, engagement in paid work, and received ration or cooked meal from Anganwadi center.

§Number of adolescent boys and girls (n) in the sample (N).

¶Adjusted for age, sex, caste, state, residence, household wealth index quintile, mother’s education, dietary score, deworming control, engagement in paid work, experience of gender discrimination within family, and discussion on physical change with parents.

††Adjusted for age, sex, religion, caste, state, household wealth index quintile, mother’s education, dietary score, deworming control, engagement in paid work, experience of sexual abuse, and discussion on physical change with parents.

††Adjusted for age, sex, religion, caste, state, residence, household wealth index quintile, mother’s education, dietary score, deworming control, engagement in paid work, experience of sexual abuse, number of friends, ever used substances, depressive symptoms, exposure to parental violence, discussion on school performance with parents and experience of gender discrimination within the family.

‡‡*P* < 0.001.

§§*P* < 0.05.

Participants who received IFA supplementation were likely to have better reading (aOR = 1.47; 95% CI = 1.07, 2.01; *P* = 0.02) and math proficiency (aOR = 1.51; 95% CI = 1.16, 1.98; *P* < 0.001) and less likely to drop out from the school (aOR = 0.72; 95% CI = 0.54, 0.96; *P* = 0.03) compared to those who did not receive IFA supplementation.

There were no significant associations between receiving deworming tablets and adolescent hemoglobin, height-for-age *z*-score, anemia, stunting, and underweight (**Table 4**). However, there was a small but negative association between receiving deworming tablets and BMI-for-age score (aMD = -0.09: 95% CI = -0.19, -0.00; *P* = 0.04). We observed no association between the interaction of deworming control and sex with hemoglobin (*P* = 0.87), BMI-for-age score (*P* = 0.77), and height-for-age score (*P* = 0.52; Table S1 in the **Online Supplementary Document**). There was also no association between receiving deworming tablets and learning outcomes (**Table 4**).

**Table 4:** Associations between deworming control and nutrition and learning outcomes in adolescent boys and girls in Uttar Pradesh and Bihar, India (2015-16 to 2018-19)

Continuous outcomes	Received deworming control, mean (SD), N = 772	Not received deworming control*, mean (SD), N = 2603	Unadjusted β† (95% CI)	Adjusted β† (95% CI)
Hemoglobin (g/dL)‡	12.36 (1.90)	12.31 (1.96)	0.05 (-0.11, 0.23)	-0.02 (-0.17, 0.12)
BMI-for-age z-score‡	-1.12 (1.05)	-0.90 (1.12)	-0.20 (-0.29, -0.11)‡‡	-0.09 (-0.19, -0.00)§§
Height-for-age z-score‡	-1.35 (0.96)	-1.37 (1.02)	0.03 (-0.05, 0.11)	0.03 (-0.05, 0.11)
**Binary outcomes**	**(n/N)**§	**(n/N)**§	**Unadjusted odds ratio**† **(95% CI)**	**Adjusted odds ratio**† **(95% CI)**
Anemia‡	84/706	305/2365	1.03 (0.71, 1.48)	1.21 (0.79, 1.84)
Stunting¶	69/644	213/2116	1.08 (0.77, 1.51)	0.90 (0.61, 1.31)
Underweight**	47/660	120/2206	1.35 (0.80, 2.26)	1.19 (0.67, 2.13)
Reading proficiency††	1229/1714	7145/9787	1.11 (0.93, 1.31)	1.10 (0.91, 1.32)
Math proficiency††	936/1714	4398/9787	1.64 (1.41, 1.90)‡‡	1.08 (0.92, 1.26)
School dropout††	411/1615	2192/7729	0.85 (0.72, 1.01)	0.89 (0.76, 1.06)

SD – standard deviation, CI – confidence interval

*Reference category for all models.

†All unadjusted and adjusted models control for sampling unit–level clustering and sampling weights.

‡Adjusted for age, sex, caste, state, residence, household wealth index quintile, mother’s education, dietary score, receiving IFA supplementation, engagement in paid work, and received ration or cooked meal from Anganwadi center.

§Number of adolescent boys and girls (n) in the sample (N).

¶Adjusted for age, sex, caste, state, residence, household wealth index quintile, mother’s education, dietary score, receiving IFA supplementation, engagement in paid work, experience of gender discrimination within family, and discussion on physical change with parents.

**Adjusted for age, sex, religion, caste, state, household wealth index quintile, mother’s education, dietary score, receiving IFA supplementation, engagement in paid work, experience of sexual abuse, and discussion on physical change with parents.

††Adjusted for age, sex, religion, caste, state, residence, household wealth index quintile, mother’s education, dietary score, receiving IFA supplementation, engagement in paid work, experience of sexual abuse, number of friends, ever used substances, depressive symptoms, exposure to parental violence, discussion on school performance with parents and experience of gender discrimination within the family.

‡‡*P* < 0.001.

§§*P* < 0.05.

**Table 5** presents the crude and adjusted associations between adolescent dietary score and nutrition status and academic outcomes. Compared to those with the lowest dietary score quartile among adolescents, the most diverse diet was associated with increased hemoglobin and improved height-for-age score. Overall, there was no evidence of the association between dietary score and BMI-for-age score, anemia, stunting, or underweight. Dietary score was significantly associated with hemoglobin among boys but not girls (*P* for interaction = 0.03) (Table S1 in the **Online Supplementary Document**). Boys from the highest quartile of dietary score were likely to have higher hemoglobin (aMD = 0.29; 95% CI = -0.05, 0.65) compared with boys from the lowest quartile of dietary score whereas there was no significant difference between the hemoglobin of the girls from the highest and lowest quartiles of dietary score (aMD = 0.17; 95% CI = -0.14, 0.49). Dietary score was significantly associated with BMI-for-age z-score among girls but not boys (p for interaction = 0.01) (Table S3 in the **Online Supplementary Document**). Girls from the highest quartile of dietary score were likely to have a higher BMI-for-age z-score compared with girls from the lowest quartile of dietary score (aMD = 0.21; 95% CI = -0.00, 0.43). There was no significant difference between the BMI-for-age z-scores of the boys from the highest and the lowest quartiles of the dietary score (aMD = 0.06; 95% CI = -0.17, 0.31). There was no interaction between dietary score and sex with the height-for-age z-score (*P* for interaction = 0.23) (Table S1 in the **Online Supplementary Document**). A higher dietary score was associated with increased odds of having higher math proficiency (aOR = for the highest quartile of dietary score 1.12; 95% CI = 1.00, 1.44; *P* for trend test = 0.05) and a lower risk of school dropout for adolescents (aOR = for the highest quartile of dietary score 0.81; 95% CI = 0.65, 1.00; *P* for trend test = 0.01). There was no statistically significant association between dietary score and reading proficiency.

**Table 5:** Associations between dietary diversity and nutrition and learning outcomes in adolescent boys and girls in Uttar Pradesh and Bihar, India (2015-16 to 2018-19)

Continuous outcomes	Dietary score quartile										
**Mean (SD)**				Unadjusted β* **(95% CI)**			Adjusted β* **(95% CI)**			***P* for trend test**
	**1^st^†, N = 1269**	**2^nd^, N = 1173**	**3^rd^, N = 713**	**4^th^, N = 220**	**2^nd^**	**3^rd^**	**4^th^**	**2^nd^**	**3^rd^**	**4^th^**	
Hemoglobin (g/dL)‡	12.16 (1.93)	12.38 (1.93)	12.46 (1.97)	12.51 (2.02)	0.21‡‡ (0.05, 0.36)	0.30§§ (0.12, 0.48)	0.32‡‡ (-0.01, 0.66)	0.09 (-0.03, 0.21)	0.20‡‡ (0.05, 0.34)	0.25‡‡ (0.01, 0.49)	0.003
BMI-for-age z-score‡	-0.99 (1.08)	-0.99 (1.10)	-0.89 (1.14)	-0.70 (1.14)	-0.001 (-0.09, 0.08)	0.09 (-0.00, 0.20)	0.28§§ (0.12, 0.43)	-0.02 (-0.10, 0.06)	0.02 (-0.08, 0.13)	0.14‡‡ (-0.00, 0.29)	0.177
Height-for-age z-score‡	-1.48 (0.97)	-1.37 (1.03)	-1.23 (1.00)	-1.07 (1.06)	0.11‡‡ (0.03, 0.19)	0.24§§ (0.15, 0.33)	0.40§§ (0.25, 0.55)	0.03 (-0.04, 0.11)	0.10‡‡ (0.01, 0.19)	0.21‡‡ (0.07, 0.37)	<0.001
**Binary outcomes**	**n/N**§				Unadjusted odds ratio* (95% CI)			Adjusted odds ratio* **(95% CI)**			
Anemia‡	157/1155	137/1068	72/646	23/202	0.92 (0.64, 1.32)	0.98 (0.66, 1.46)	0.91 (0.51, 1.61)	0.87 (0.59, 1.27)	0.92 (0.61, 1.37)	0.77 (0.41, 1.43)	0.420
Stunting¶	132/1016	95/949	39/601	16/194	0.85 (0.61, 1.20)	0.47§§ (0.30, 0.73)	0.70 (0.33, 1.50)	0.92 (0.63, 1.33)	0.54‡‡ (0.33, 0.87)	0.87 (0.40, 1.91)	0.079
Underweight**	56/1078	60/991	38/601	13/196	1.23 (0.78, 1.94)	1.62 (0.89, 2.94)	1.28 (0.59, 2.79)	1.20 (0.75, 1.91)	1.57 (0.90, 2.73)	1.17 (0.51, 2.68)	0.143
Reading proficiency**	2591/3964	3017/4068	1894/2447	872/1022	1.37§§ (1.21, 1.54)	1.60§§ (1.35, 1.90)	2.47§§ (1.83, 3.33)	1.13 (0.98, 1.29)	1.06 (0.88, 1.26)	1.19 (0.86, 1.63)	0.237
Math proficiency††	1451/3964	1906/4068	1348/2447	629/1022	1.34§§ (1.18, 1.53)	1.72§§ (1.48, 1.99)	2.06§§ (1.66, 2.57)	1.10‡‡ (1.00, 1.28)	1.20‡‡ (1.02, 1.40)	1.12‡‡ (1.00, 1.44)	<0.05
School dropout††	962/2974	956/3336	498/2124	187/910	0.87 (0.75, 1.01)	0.68§§ (0.57, 0.81)	0.64§§ (0.51, 0.80)	0.94 (0.79, 1.12)	0.81‡‡ (0.67, 0.99)	0.81 (0.65, 1.00)	<0.05

SD – standard deviation, CI – confidence interval

*All unadjusted and adjusted models control for sampling unit–level clustering and sampling weights.

†Reference category for all models.

‡Adjusted for age, sex, caste, state, residence, household wealth index quintile, mother’s education, receiving IFA supplementation, deworming control, engagement in paid work, and received ration or cooked meal from Anganwadi center.

§Number of adolescent boys and girls (n) in the sample (N).

¶Adjusted for age, sex, caste, state, residence, household wealth index quintile, mother’s education, receiving IFA supplementation, deworming control, engagement in paid work, experience of gender discrimination within family, and discussion on physical change with parents.

**Adjusted for age, sex, religion, caste, state, household wealth index quintile, mother’s education, receiving IFA supplementation, deworming control, engagement in paid work, experience of sexual abuse, and discussion on physical change with parents.

††Adjusted for age, sex, religion, caste, state, residence, household wealth index quintile, mother’s education, IFA supplementation, deworming control, engagement in paid work, experience of sexual abuse, number of friends, ever used substances, depressive symptoms, exposure to parental violence, discussion on school performance with parents and experience of gender discrimination within the family.

‡‡*P* < 0.001.

§§*P* < 0.05.

## DISCUSSION

We found no association between receiving IFA supplementation with nutritional outcomes, however, we found that the association of receiving IFA supplementation with hemoglobin and height-for-age z-score differed by sex. Deworming was negatively associated with BMI-for-age score, and dietary scores were positively associated with hemoglobin and height-for-age score. Receiving IFA supplementation was positively associated with learning outcomes while a higher dietary score was associated with better math proficiency and a reduced risk of school dropout. We also found that the coverage of adolescents with IFA supplementation and deworming was poor; it was poorer among boys than girls.

The association of female sex with increased risk of anemia, and higher BMI- and lower height-for-age scores are in line with previously reported studies [34-36]. In the NFHS-3 and NFHS-4, anemia was consistently higher in girls (aged 15-19 years) compared to same-aged boys, and the mean BMI-for-age *z*-score was higher among girls than boys [34]. The observed higher risk for anemia among Indian adolescent girls suggests that they may experience greater exposure to risk factors for chronic nutritional deficiency and undernutrition compared with their male peers. For example, adolescent girls are likely to have a low intake of iron-rich foods, and erratic eating habits due to discriminatory societal norms and practices, although they have increased requirements for iron during the growth spurt, which are further increased by menstrual losses [37]. Additionally, poor dietary intake by girls, means that they are more likely to become stunted compared to boys. In line with our findings, the higher risk of underweight for adolescent boys, compared to girls is documented in many studies in India [38-41]. For example, a seminal study of 12 124 adolescents across nine Indian states found that 53.1% of boys and 39.5% of the girls were underweight [38]. Irrespective of the sample size, rural or urban residence, these studies report underweight in adolescent boys being worse than that in girls, and this has been attributed to energy intake below the recommended level for boys [38-40], whereas girls were less likely to be underweight because they suffer more from stunting.

Although we observed no association between receiving IFA supplementation and nutrition and growth outcomes in this study, this does not imply that the government-run weekly IFA supplementation program is a failure in the states of Uttar Pradesh and Bihar. We found indicative evidence of higher hemoglobin among those girls who received IFA supplements than those who did not receive it and a higher height-for-age *z*-score among those boys who received IFA supplements than those who did not receive it. In a year-long community-based before-and-after study of IFA supplementation in Gujarat, the prevalence of anemia decreased from 79.5% to 58.0% among adolescent girls and from 64.0% to 39.0% among adolescent boys, while the mean increase in hemoglobin was 1.5g/dL and 1.3 g/dL among boys and girls, respectively [14]. Additionally, an evaluation of a year-long weekly IFA supplementation program targeting adolescent girls from 52 districts of 13 states in India found a 24% average reduction in the prevalence of anemia [42]. Additionally, in a study of 203 adolescent girls from Gujarat, India (aged 10-18 years), IFA supplementation was associated with a significant increase in weight (0.83kg) compared with no supplementation [11]. Similarly, in a study of 254 girls in India, significant increments in BMI were observed when the girls were treated with IFA tablets either twice a week or daily [12]. Further, a systematic review of 31 studies of IFA supplementation from around the world, either as a standalone intervention or in combination with other micronutrients, suggests an overall significant reduction in anemia among adolescents (relative risk 0.69; 95% CI = 0.62, 0.76) [43]. For example, Ghana has also rolled out a nationwide IFA Supplementation program in 2019-2020. School-going adolescent girls in Ghana receive holistic nutrition education with intermittent weekly IFA supplementation. An evaluation of this program estimated a reduction in the prevalence of anemia from 25% to 19.5% over 1 school year, but adherence, defined as the number of tablets consumed each week of the school year after program launch (30-36 week depending on the school), was suboptimal (approximately half-consumed ≤15 tablets) [44]. Nevertheless, both India and Ghana’s national programs are experiencing bottlenecks to the optimal coverage and it would be important to identify the contextual and structural barriers to adherence and implement strategies to mitigate these barriers.

Notably, our study found a positive association between receiving IFA supplementation and learning outcomes. Adolescents who received IFA supplementation were more likely to have better reading and math proficiency and a reduced risk of school dropout compared to those who did not receive IFA supplementation, possibly resulting in some improvements in iron status even though it was not sufficient to lead to reduced iron deficiency anemia. Similar findings were observed in an experimental study, where daily IFA supplementation was associated with higher cognitive functioning compared among Indian adolescent girls [12]. There is ample support to the argument that improved cognition may lead to better academic performance, which may be an incentive for adolescents to remain in school [8,45]. In contrast, nutritional deficiencies can affect the cognitive development of adolescents [46]. Cognitive growth depends on micronutrients such as Vitamin B12, folate, and thiamine are important for neural pathways, and their deficiency has been linked to impaired episodic memory and language issues [47]. Iron is required for oligodendrocyte growth and neurotransmitter production, and deficiency affects cognition, memory, and social and motor development [46]. However, the evidence for nutrition and academic achievement remains weak in developing countries and warrants further investigation.

There could be various explanations for our observed findings of no association of receiving IFA supplementation and deworming with nutrition and growth status of adolescents. First, the coverage of IFA supplementation and deworming was very low among study participants. It may also be due to low compliance, that is, the adolescents were given the IFA supplementation and deworming tablets but they did not consume them. Several studies have reported adolescents’ non-compliance with IFA supplementation because of its taste and side effects [10,14,48]. For example, a cross-sectional study of 240 adolescents in India reported that girls perceived that IFA tablets caused weight gain, could have side effects such as bad taste, abdominal pain, and giddiness, while boys considered that IFA was not necessary, and could have side effects such as stomach pain and nausea [48].

Second, there may have been many obstacles in the supervised administration of weekly IFA supplementation through schools and frontline health workers. Fifty-two doses of IFA supplementation are required to be administered to adolescents every year under the weekly IFA supplementation program of India. However, under supervised administration through schools, only about 28 doses can be distributed due to weeks of summer holidays, winter vacations, government holidays, and examination schedules. On the other hand, voluntary frontline health workers have been given the responsibility to distribute the IFA and deworming tablets to out-of-school adolescents. These frontline health workers distribute the IFA supplementation and deworming tablets alongside their other competing priorities and existing burden of work, which keeps on increasing with newer responsibilities assigned by the Ministry of Health and Family Welfare and other ministries [10].

Third, the IFA supplementation program has not yet operationalized the supplementary component of the ‘test and treat’ strategy, that is, screening for moderate/severe anemia and referring these cases to an appropriate health facility [10]. At the national and regional levels, barriers and facilitators have been identified in terms of resources, capacity, and training, enabling environment, and cross-sector collaborations [14].

Fourth, the implementation of programs in the community is influenced by different factors, including caregivers and other community members' support, resistance, or indifference, as well as cultural norms and media, as well as educational activities [10]. Adolescents' likelihood of consuming IFA tablets may be affected by their individual and societal knowledge, attitudes, perceptions, and experiences of anemia risk and IFA together with impacts associated with physical and cognitive changes associated with taking them [14,48].

Finally, inadequate efforts are directed towards effective behavior change communication for improving dietary intake and taking actions to prevent intestinal worm infestation among adolescents [49,50]. To close this policy-to-practice gap, evidence-based and implementation science strategies should be used. For example, teachers and frontline health workers should act to improve adolescents’ knowledge, attitudes, and beliefs about IFA supplementation and ensure adherence beyond mere distribution. Our findings of a small though not statistically significant association between receiving IFA and hemoglobin concentrations does not preclude the possibility that supplementation was associated with reduced risk of iron deficiency that was not severe enough to lead to anemia, but may be important for improving adolescent development and school performance. Unfortunately, we have not measured iron status in this population, and this would be important to pursue in future studies.

Our findings highlight the beneficial relationships of diverse dietary intake on nutritional and growth status for adolescents in terms of improved hemoglobin levels and height-for-age z-score, which corroborates the findings of limited available literature [50-52]. Particularly, we found that boys with a more diverse diet were likely to have high hemoglobin, and girls having a more diverse diet were likely to have a high BMI-for-age score. In a cross-sectional study of 189 adolescents (aged 13-17 years) from Maharashtra and Odisha states in India, higher household dietary diversity was associated with higher BMI- and height-for-age z-scores of the adolescents [51]. Higher dietary diversity is associated with higher macro-and micronutrient intake in adolescents that is, individuals consuming more diverse diets are more likely to meet their nutrient needs [53]. Dietary diversity has been shown in adults to be predictive of micronutrient adequacy [48,54]. In developing countries, diversifying diets have been used as a key strategy to enhance dietary intake and address nutritional deficiencies [46,54] and there is widespread recognition that low dietary diversity is associated with chronic nutritional deficiencies [20]. However, consuming a more diverse diet is beyond the reach of most households in India because of limited livelihood and income opportunities, and increasing food prices. As a result, households switch to cheaper and less nutritious foods and compromise the quality of the food consumed.

Our results also show a significant potential for improving dietary diversity to enhance adolescent learning outcomes. We noted that high dietary diversity had a beneficial association with math proficiency skills. With increasing enrolments in primary and secondary education in India [55], schools can play a critical role in shifting adolescents’ perceptions of food and enhancing access to healthful and diverse foods. Amid the litany of new education policies that emphasizes innovation and new methods, nutrition education, school gardens, homestead gardens stand out as a low-tech change. Our findings also call for strengthening the present mid-day meal program by expanding its coverage to a universal school lunch program covering school-going and out-of-school adolescents, available across both government and private schools, with more variety, and greater micro-nutrient coverage.

### Strengths and limitations of this analysis

Much of the existing evidence is derived from cross-sectional demographic and health surveys among adolescent girls and adult women of reproductive age who have had a previous pregnancy or small-scale school-based surveys. In this context, our study has several strengths. First, it involves a large sample size across the full adolescent age spectrum from two large states of India. Second, our analysis is based on the longitudinal data of the adolescents, which accurately ascertains the timing of exposures with the studied outcomes. Third, our sample included both school-going and out-of-school adolescents, whereas most previous adolescent health surveys conducted in India have only included school-going adolescents and therefore, are less generalizable. Finally, our sample included both adolescent boys and girls, whereas most previous intervention studies of IFA supplementation and dietary diversity are conducted with only adolescent girls. Hence, our findings contribute to developing country literature.

There are also some limitations to this study. In the present study, data on compliance of IFA supplementation and deworming was not available, which could have been useful in further unfolding the results. Second, the food groups and the recall period used to measure the dietary diversity were not as comprehensive as recommended by the FAO [24]. Finally, we controlled for different factors affecting the nutritional status and educational outcomes in the multivariable models; however, the study did not collect data on some potential confounders such as the physical activity, the prevalence of infection and parasitic infestation, diarrhea episodes, and cultural and gender norms were not collected.

## CONCLUSION

The absolute burden of poor nutritional outcomes including anemia, stunting, and underweight was high among adolescents in Uttar Pradesh and Bihar, in India. Girls were at increased risk of anemia, had lower hemoglobin and height-for-age score, and higher BMI-for-age score, compared to boys. Girls also had poorer reading and math proficiency and a higher risk of school dropout than boys. Receiving IFA supplementation was positively associated with hemoglobin among girls and height-for-age score among boys. Beneficial associations were observed between receiving IFA supplementation and adolescent learning outcomes. Diverse dietary intake was positively associated with hemoglobin among boys, BMI-for-age score among girls, and adolescents’ height-for-age score, math proficiency, and a lower risk of school dropout. Strengthening the existing universal weekly IFA supplementation program with gender-specific strategies and dietary interventions may be required to improve the nutritional status of adolescents in these states as well as in India. No evidence of an association between receiving IFA supplementation and hemoglobin among adolescent boys warrants further investigation. Investing in the nutritional status of the largest generation of adolescents will bring benefits to their health today, their well-being for decades to come, and for the next generation.

## Additional material

Online Supplementary Document

## References

[1] Galloway R. Global nutrition outcomes at ages 5 to 19. (Chapter 3 in eds) Bundy D, De’Silva N, Horton S, Hamison DT, Patton GC. Child and adolescent health development. Disease Control Priorities 3rd Edition. Washington DC: World Bank; 2017. 30212125

[2] Christian P Smith ER Adolescent undernutrition: global burden, physiology, and nutritional risks. Ann Nutr Metab. 2018;72:316-28. 10.1159/000488865 29730657

[3] Patton GC Sawyer S Santelli JS Ross DA Afifi R Allen NB Our future: a Lancet commission on adolescent health and wellbeing. Lancet. 2016;387:2423-78. 10.1016/S0140-6736(16)00579-1 27174304 PMC5832967

[4] United Nations. World Population Prospects: The 2017 Revision, DVD Edition. 2017.

[5] India State-Level Disease Burden Initiative Malnutrition Collaborators The burden of child and maternal malnutrition and trends in its indicators in the states of India: The Global Burden of Disease Study 1990–2017. Lancet Child Adolesc Health. 2019;3:855-70. 10.1016/S2352-4642(19)30273-1 31542357 PMC6839043

[6] NCD Risk Factor Collaboration (NCD–RisC) Worldwide trends in body-mass index, underweight, overweight, and obesity from 2005-2016: a pooled analysis of 2416 population-based measurement studies in 128.9 million children, adolescents, and adults. Lancet. 2017;390:2627-42. 10.1016/S0140-6736(17)32129-3 29029897 PMC5735219

[7] Chaparro CM Suchdev PS Anemia epidemiology, pathophysiology, and etiology in low- and middle-income countries. Ann N Y Acad Sci. 2019;1450:15-31. 10.1111/nyas.14092 31008520 PMC6697587

[8] World Health Organization. Global health risks: mortality and burden of disease attributable to selected major risks. Geneva: WHO; 2009.

[9] Petry N Olofin I Hurrell R Boy E Wirth JP Moursi M The proportion of anemia associated with iron deficiency in low, medium, and high human development index countries: a systematic analysis of national surveys. Nutrients. 2016;8:693-710. 10.3390/nu8110693 27827838 PMC5133080

[10] Kapil U Kapil R Gupta A National Iron Plus Initiative: current status and future strategy. Indian J Med Res. 2019;150:239-47. 10.4103/ijmr.IJMR_1782_18 31719294 PMC6886130

[11] Kanani SJ Poojara RH Supplementation with iron and folic acid enhances growth in adolescent Indian girls. J Nutr. 2000;130:452S-5S. 10.1093/jn/130.2.452S 10721926

[12] Sen A Kanani SJ Intermittent iron folate supplementation: impact on hematinic status and growth of schoolgirls. ISRN Hematol. 2012;2012:482153. 10.5402/2012/482153 22919508 PMC3412096

[13] Aguayo VM Paintal K Singh G The adolescent girls’ Anaemia Control Programme: a decade of programming experience to break the intergenerational cycle of malnutrition in India. Public Health Nutr. 2013;16:1667-76. 10.1017/S1368980012005587 23343620 PMC10271399

[14] Shah SP Shah P Desai S Modi D Desai G Arora H Effectiveness and feasibility of weekly iron and folic acid supplementation to adolescent girls and boys through peer educators at community-level in the tribal area of Gujarat. Indian J Community Med. 2016;41:158-61. 10.4103/0970-0218.173498 27051093 PMC4799641

[15] Varghese JS Swaminathan S Kurpad AV Thomas T Demand and supply factors of iron-folic acid supplementation and its association with anemia in Northern Indian pregnant women. PLoS One. 2019;14:e0210634. 10.1371/journal.pone.0210634 30699167 PMC6353128

[16] Grebmer VK, Bernstein J, Hossain N, Brown T, Prasai N, Yohannes Y, et al. Global Hunger Index: The inequalities of hunger: Synopsis. Washington, DC, Bonn, and Dublin: International Food Policy Research Institute, Welthungerhilfe, and Concern Worldwide; 2017.

[17] Sachdev H Gera T Nestel P Effect of iron supplementation on mental and motor development in children: a systematic review of randomized controlled trials. Public Health Nutr. 2005;8:117-32. 10.1079/PHN2004677 15877905

[18] Low M Farrell A Biggs BA Pasricha SR Effects of daily iron supplementation in primary-school-aged children: systematic review and meta-analysis of randomized controlled trials. CMAJ. 2013;185:E791-802. 10.1503/cmaj.130628 24130243 PMC3832580

[19] Falkingham M Abdelhamid A Curtis P Fairweather-Tait S Dye L Hooper L The effects of oral iron supplementation on cognition in older children and adults: a systematic review and meta-analysis. Nutr J. 2010;9:4-19. 10.1186/1475-2891-9-4 20100340 PMC2831810

[20] Arimond M Ruel MT Dietary diversity is associated with child nutritional status: evidence from 11 demographic and health surveys. J Nutr. 2004;134:2579-85. 10.1093/jn/134.10.2579 15465751

[21] UNICEF. India Office. Adolescent nutrition: investing in an age of opportunity to break cycles of poverty and inequity. Available: https://www.unicef.org/india/what-we-do/adolescent-nutrition. Accessed: 10 January 2019.

[22] Population Council. Understanding the lives of adolescents and young adults (UDAYA) 2015–2020. www.projectudaya.in.

[23] ASER Centre. Annual status of education report (Rural) 2016, Provisional. New Delhi: ASER, 2017. Available: http://www.asercentre.org/p/289.html 6. Accessed: 10 January 2019.

[24] Kennedy G, Ballard T, Dop MC. Guidelines for Measuring Household and Individual Dietary Diversity. Rome: Food and Agriculture Organization; 2010.

[25] World Health Organization. Hemoglobin concentrations for the diagnosis of anemia and assessment of severity. Geneva: WHO; 2011.

[26] Butte NF Garza C de Onis M Feasibility of international growth standards for school-aged children and adolescents. J Nutr. 2007;137:153-7. 10.1093/jn/137.1.153 17182818

[27] de Onis MA Onyango W Borghi E Development of a WHO growth reference for school-aged children and adolescents. Bull World Health Organ. 2007;85:660-7. 10.2471/BLT.07.043497 18026621 PMC2636412

[28] Chai J Fink G Kaaya S Danaei G Fawzi W Ezzati M Association between intimate partner violence and poor child growth: results from 42 demographic and health surveys. Bull World Health Organ. 2016;94:331-9. 10.2471/BLT.15.152462 27147763 PMC4850526

[29] Ackerson LK Subramanian SV Domestic violence and chronic malnutrition among women and children in India. Am J Epidemiol. 2008;167:1188-96. 10.1093/aje/kwn049 18367471 PMC2789268

[30] O’Neil A Quirk SE Housden S Brennan SL Williams LJ Pasco JA Relationship between diet and mental health in children and adolescents: a systematic review. Am J Public Health. 2014;104:e31-42. 10.2105/AJPH.2014.302110 25208008 PMC4167107

[31] Kroenke K Spitzer RL Williams JB The PHQ–9: validity of a brief depression severity measure. J Gen Intern Med. 2001;16:606-13. 10.1046/j.1525-1497.2001.016009606.x 11556941 PMC1495268

[32] Ganguly S Samanta M Roy P Chatterjee S Kaplan DW Basu B Patient Health Questionnaire-9 as an effective tool for screening of depression among Indian adolescents. J Adolesc Health. 2013;52:546-51. 10.1016/j.jadohealth.2012.09.012 23299020

[33] Groenwold RHH White IR Donders ART Carpenter JR Altman DG Moons KGM Missing covariate data in clinical research: when and when not to use the missing-indicator method for analysis. CMAJ. 2012;184:1265-9. 10.1503/cmaj.110977 22371511 PMC3414599

[34] Bhargava M Bhargava A Ghate SD Rao RSP Nutritional status of Indian adolescents (15-19 years) from National Family Health Surveys 3 and 4: revised estimates using WHO growth reference. PLoS One. 2020;15:e0234570. 10.1371/journal.pone.0234570 32970776 PMC7514009

[35] Soman SK Arrekal B Murli AJ Varghese RG Adolescent anemia its prevalence and determinants: a cross-sectional study from south Kerala, India. Int J Community Med Public Health. 2017;4:2750-6. 10.18203/2394-6040.ijcmph20173318

[36] Pal A Pari AK Sinha A Prevalence of undernutrition and associated factors: a cross-sectional study among rural adolescents in West Bengal, India. Int J Pediatr Adolesc Med. 2017;4:9-18. 10.1016/j.ijpam.2016.08.009 30805494 PMC6372453

[37] Ministry of Health and Family Welfare, Government of India. Guidelines for Control of Iron Deficiency Anaemia. New Delhi: GoI; 2013.

[38] Venkaiah K Damayanti K Nayak MU Vijayaraghvan K Diet and nutritional status of rural adolescents in India. Eur J Clin Nutr. 2002;56:1119-25. 10.1038/sj.ejcn.1601457 12428178

[39] Das DK Biswas R Nutritional status of adolescent girls in a rural area of North 24 Parganas district, West Bengal. Indian J Public Health. 2005;49:18-21. 15989155

[40] Medhi GK Hazarika NC Mahanta J Nutritional status of adolescents among tea garden workers. Indian J Pediatr. 2007;74:343-7. 10.1007/s12098-007-0057-3 17476077

[41] Banerjee S Dias A Shinkre R Patel V Undernutrition among adolescents: a survey in five secondary schools in rural Goa. Natl Med J India. 2011;24:8-11. 21608350 PMC4991764

[42] Malhotra S Yadav K Kusuma YS Sinha S Yadav V Pandav CS Challenges in scaling up successful public health interventions: lessons learned from resistance to a nationwide rollout of the weekly iron-folic acid supplementation program for adolescents in India. Natl Med J India. 2015;28:81-5. 26612152

[43] Salam RA Hooda M Das JK Arshad A Lassi ZS Middleton P Interventions to improve adolescent nutrition: a systematic review and meta-analysis. J Adolesc Health. 2016;59:S29-39. 10.1016/j.jadohealth.2016.06.022 27664593 PMC5026685

[44] Gosdin L, Sharma AJ, Tripp K, Amoaful EF, Mahama AB, Selenje L, et al. Barriers to and facilitators of Iron and Folic Acid supplementation within a school-based integrated nutrition and health promotion program among Ghanaian adolescent girls. CDN. 2020; 10.1093/cdn/nzaa135. 10.1093/cdn/nzaa135 PMC7467268 32914043

[45] Peng P Kievit RA The development of academic achievement and cognitive abilities: a bidirectional perspective. Child Dev Perspect. 2020;14:15-20. 10.1111/cdep.12352 PMC7613190 35909387

[46] Lassi Z, Moin A, Bhutta Z. Nutrition in middle childhood and adolescence (Chapter 11 in eds) Bundy D, De’Silva N, Horton S, Hamison DT, Patton GC. Child and adolescent health development. Disease Control Priorities 3rd Edition. Washington DC: World Bank; 2017.

[47] Katz DL, Friedman RSC. Diet and cognitive function. Nutrition in clinical practice: a comprehensive, evidence-based manual for the practitioner. Philadelphia: Lippincott Williams & Wilkins; 2008.

[48] Priya SH Datta SS Bahurupi YA Narayan KA Nishanthini N Ramya MR Factors influencing weekly iron-folic acid supplementation program among schoolchildren: where to focus our attention? Saudi J Health Sci. 2016;5:28-33. 10.4103/2278-0521.182863

[49] Gupta S Pingali PL Pinstrup-Andersen P Women’s empowerment and nutrition status: the case of iron deficiency in India. Food Policy. 2019;88:101763. 10.1016/j.foodpol.2019.101763

[50] Radhika MS Swetha B Naveen KB Bala KN Laxmaiah A Dietary and nondietary determinants of nutritional status among adolescent girls and adult women in India. Ann N Y Acad Sci. 2018;1416:5-17. 10.1111/nyas.13599

[51] Nithya DJ Bhavani RV Dietary diversity and its relationship with nutritional status among adolescents and adults in rural India. J Biosoc Sci. 2018;50:397-413. 10.1017/S0021932017000463 28967344

[52] Agustina R Nadiya K Andini EA Setianingsih AA Sadariskar AA Prafiantini E Associations of meal patterning, dietary quality and diversity with anemia and overweight-obesity among Indonesian school-going adolescent girls in West Java. PLoS One. 2020;15:e0231519. 10.1371/journal.pone.0231519 32324775 PMC7179884

[53] World Health Organization. Preparation and use of food-based dietary guidelines. WHO/NUT/96.6. Geneva: Nutrition Programme, WHO; 1996.

[54] Gupta S Sunder N Pingali PL Market access, production diversity, and diet diversity: evidence from India. Food Nutr Bull. 2020;41:167-85. 10.1177/0379572120920061 32522130

[55] Ministry of Human Resource and Development, Government of India. Educational Statistics at a glance, 2018. Available: https://www.mhrd.gov.in/sites/upload_files/mhrd/files/statistics-new/ESAG-2018.pdf Accessed: 15 March 2019.

