# Supplementary material for: Association of iron supplementation and dietary diversity with nutritional status and learning outcomes among adolescents: Results from a longitudinal study in Uttar Pradesh and Bihar, India
Source: J Glob Health. 2021 Jul 24;11:04037. doi: 10.7189/jogh.11.04037 (PMC8325919; doi:10.7189/jogh.11.04037)
Supplement: Online Supplementary Document [file jogh-11-04037-s001.pdf]

Supplement Table 1: Number of participants who completed interviews and anthropometric assessments in UDAYA project surveys in Uttar Pradesh and Bihar, India

Results of interviews and anthropometric assessments	Number
Number of 10-19 years old unmarried male and female adolescents and 15-19 years old married females who completed the first survey interview in 2015-16	20,589
Number of 10-19 years old unmarried male and female adolescents and 15-19 years old married females who completed the second survey interview in 2018-19	16, 292
Number of 10-19 years old unmarried male and female adolescents who completed the first survey interview in 2015-16	15, 388
Number of 10-19 years old unmarried male and female adolescents who completed the second survey interview in 2018-19	12, 035
Number of 10-19 years old unmarried male and female adolescents who completed round 1 & 2 interviews and considered for analyses of learning outcomes (i.e., reading and math proficiency)	12, 035
Number of 10-19 years old unmarried male and female adolescents who completed round 1 & 2 interviews and included for analysis of school dropout outcome based on school-enrolment status	9, 344
Number of anthropometric and biomarker assessments completed with a subsample of 10-19 years old unmarried male and female adolescents in the first round of survey in 2015-16	5,987
Number of anthropometric and biomarker assessments completed with a subsample of 10-19 years old unmarried male and female adolescents in the second round of survey in 2018-19	3,382
Number of 13-19 years old unmarried male and female adolescents during the second round of survey considered for growth and nutritional outcomes analyses	3,071

Supplement Table 2: Percentage of adolescents who consumed selected food items by frequency of consumption in Uttar Pradesh and Bihar in 2015–16

Type of food (in %)	Girls (N=7607)				Boys (N=4428)			
	**Daily**	**Weekly**	**Occasionally**	**Never**	**Daily**	**Weekly**	**Occasionally**	**Never**
Pulses/beans	46.17	43.87	9.37	0.59	48.78	45.01	6.03	0.18
Dark green vegetables	16.45	59.66	22.56	1.34	13.37	54.61	29.31	2.71
Other vegetables	87.92	10.17	1.80	0.11	78.93	18.02	2.94	0.11
Fruits	9.81	31.92	57.50	0.78	8.69	30.33	60.21	0.77
Eggs	1.92	28.01	38.83	31.23	6.14	31.82	41.12	20.91
Meat/poultry	0.76	22.74	40.96	35.53	0.77	20.37	51.36	27.51
Fish & seafood	0.26	18.65	39.50	41.58	0.81	16.60	48.08	34.51
Milk & milk products	32.84	25.61	36.66	4.89	47.56	24.23	25.05	3.16

Supplement Table 3: *P*-values for the association of interaction between sex and exposure variable (i.e., receiving IFA supplementation, deworming control, or dietary score) with continuous outcomes

Outcomes	*P*-value for interaction between IFA supplementation and sex	*P*-value for interaction between deworming and sex	*P*-value for interaction between dietary score and sex
Hemoglobin (g/dL) ††	P=0.09	P=0.87	P<0.05
BMI−for−age z−score††	P=0.88	P=0.77	P<0.05
Height−for−age z−score††	P=0.06	P=0.52	P=0.23
